# CRISPR RNA-Guided Transposases Facilitate Dispensable Gene Study in Phage

**DOI:** 10.3390/v16030422

**Published:** 2024-03-09

**Authors:** Yanmei Liu, Zizhen Liang, Shuting Yu, Yanrui Ye, Zhanglin Lin

**Affiliations:** 1 School of Biology and Biological Engineering, South China University of Technology, Guangzhou 510006, China; 202010108468@mail.scut.edu.cn (Y.L.); 201920146292@mail.scut.edu.cn (Z.L.); 202020148762@mail.scut.edu.cn (S.Y.); 2 Shenzhen Institute of Synthetic Biology, Shenzhen Institutes of Advanced Technology, Chinese Academy of Sciences, Shenzhen 518055, China

**Keywords:** *Pseudomonas aeruginosa* phage, *V. cholerae* Tn*6677* transposon, in vivo transposon insertion

## Abstract

Phages provide a potential therapy for multi-drug-resistant (MDR) bacteria. However, a significant portion of viral genes often remains unknown, posing potential dangers. The identification of non-essential genes helps dissect and simplify phage genomes, but current methods have various limitations. In this study, we present an in vivo two-plasmid transposon insertion system to assess the importance of phage genes, which is based on the *V. cholerae* transposon Tn*6677*, encoding a nuclease-deficient type I-F CRISPR–Cas system. We first validated the system in *Pseudomonas aeruginosa* PAO1 and its phage S1. We then used the selection marker AcrVA1 to protect transposon-inserted phages from CRISPR-Cas12a and enriched the transposon-inserted phages. For a pool of selected 10 open-reading frames (2 known functional protein genes and 8 hypothetical protein genes) of phage S1, we identified 5 (2 known functional protein genes and 3 hypothetical protein genes) as indispensable genes and the remaining 5 (all hypothetical protein genes) as dispensable genes. This approach offers a convenient, site-specific method that does not depend on homologous arms and double-strand breaks (DSBs), holding promise for future applications across a broader range of phages and facilitating the identification of the importance of phage genes and the insertion of genetic cargos.

## 1. Introduction

Bacteriophages (phages)—viruses that specifically infect bacteria—are highly abundant in the biosphere, numbering up to 10^31^ [1,2]. With the rapid development of multi-drug-resistant (MDR) bacteria, phages have gained significance as a possible therapy due to their host specificity and self-amplifying features [3]. For safety, therapeutic phages are desirable to exclude toxin genes, virulence factors, and antibiotic resistance genes [4,5], and it is preferable for phages to carry a minimal number of genes with unknown functions to avoid potential dangers.

However, a substantial portion of the viral genetic information remains unknown, with 75% of the more than 2.79 million viral genes cataloged lacking identified functions and without any corresponding homologous genes found in public databases [6]. Even for the well-studied model phage T4, there is a significant gap in knowledge, as 45% of its genes are of unknown function [7]. Identifying non-essential genes for phage would aid in the analysis of phage genomes [5,8], and current functional investigation methods include homologous recombination, CRISPR-Cas, and complete genome reconstruction in non-host systems [9]. However, these approaches face various limitations. For homologous recombination, simultaneous serial insertions are challenging due to the specification of editing sites by homologous arms [10]. Additionally, short homologous arms result in low efficiency, but long arms may contain harmful phage genes [9]. Although efficiency can be improved by integrating CRISPR-Cas systems, this method risks causing cell death through double-strand breaks (DSBs) [11,12,13]. Complete genome reconstruction in non-host systems allows for genome design flexibility, but these reconstructed genomes often fail to reboot [14,15,16,17]. These emphasize the need for new phage genome engineering methods to identify the importance of phage genes.

Transposon insertion has, in the past, been used as a tool for phage gene characterization. It relies on randomly in vitro transposition using MuA transposase, followed by electroporation into host cells to identify mutant phages and determine transposon insertion sites. This method has only been applied to a few phages and its efficiency is mostly constrained by the transformation capacity of the host bacteria [18,19,20]. Recently, Klompe et al. reported a site-specific, programmable system for in vivo transposon insertion using the *V. cholerae* transposon Tn*6677*, which encodes a nuclease-deficient type I-F CRISPR–Cas system [21,22,23]. The system employs three plasmids: pQCascade, pTnsABC, and pDonor. pQCascade encodes the *tniQ-cas8-cas7-cas6* operon (QCascade) alongside a synthetic CRISPR array; thereby, the Qcascade complex surveys and selectively binds to target DNA sites guided by crRNA. pTnsABC carries the *tnsA-tnsB-tnsC* operon, where the TnsA and TnsB transposase catalyze DNA insertion, and TnsC ATPase regulates target site selection. pDonor includes a mini-transposon donor containing the DNA cargo [10,21,24]. DNA integration occurs ~50 bp from the 3′ edge of the crRNA target site with a 5-bp target-site duplication (TSD). The transposon insertion system proved effective in *E. coli*, *Klebsiella oxytoca*, and *Pseudomonas putida* [10]. This success has prompted us to apply this system to explore phage genomes.

Given the prevalence of *Pseudomonas aeruginosa* (PA) as a critical antibiotic-resistant pathogen [25,26] and the need for an alternative treatment method, we chose a *P. aeruginosa* phage S1, which was isolated and purified for our study, for transposon insertion [25]. For simplicity, we reduced the three-plasmid transposon insertion system to two plasmids, validating the viability of the two-plasmid system on PAO1 and phage S1. We then used the selection marker AcrVA1 to protect the transposon-inserted phages from being eradicated, thereby enriching this group of phages. We selected 10 open-reading frames (*orf*s) from S1 and identified 5 as indispensable genes and the other 5 as dispensable genes. This study successfully demonstrates the feasibility of using the in vivo transposon insertion system to explore the importance of phage gene, providing new insights into a convenient, site-specific method that does not depend on homologous arms and DSBs.

## 2. Materials and Methods

### 2.1. Phage, Strains, and Media

The *P. aeruginosa* phage vB_PaeM_SCUT-S1 (S1, GenBank MK340760) served as the model organism for phage transposon insertion studies, and *P. aeruginosa* PAO1 was the host strain. *P. aeruginosa* PAO1 *phzM::lacZ* was used for transposon insertion test [27]. *E. coli* DH5α was employed for plasmid cloning. All strains were cultured in lysogeny broth (LB) medium at 37 °C. Tetracycline (10 μg/mL final concentration for *E. coli*, and 100 μg/mL for *P. aeruginosa*) or gentamicin (10 μg/mL for *E. coli*, and 50 μg/mL for *P. aeruginosa*) was added to the medium when required. Transposon insertion experiments were conducted using the overlay agar method with LB containing 2 mM CaCl_2_. For the top and the base agar, 0.6% and 1.5% agar were used, respectively. Plasmids pQCascade_entry (BsaI-stuffer), pTnsABC, and pDonor [21] were purchased from Addgene (numbered 130633 to 130635). Plasmids pCas12a-λRed and pCrRNA-V were stocked in our lab [27]. KOD FX DNA polymerase from TOYOBO Biotechnology (Dalian, China) was used for all cloning procedures. Restriction enzymes, T4 polynucleotide kinase, T4 DNA ligase, and Gibson assembly reagents [28] were purchased from New England Biolabs, Inc. (Beverly, MA, USA). DNA markers were acquired from Takara Bio Inc. (Dalian, China). All chemical reagents were of analytic grade and obtained from Sigma-Aldrich (St. Louis, MO, USA) or Sangon Biotechnology (Shanghai, China). The information about all the phage, bacterial strains, and plasmids used in this study is listed in Table S2, all spacer sequences are listed in Table S3, and all primers are listed in Table S4.

### 2.2. Plasmid Construction

Construction of pQCascade-TnsABC based on pCas12a-λRed [21,27]. The DNA fragment carrying P_J23119_ and QCascade was amplified from plasmid pQCascade_entry (BsaI-stuffer) using primers ZZ21038/039; the DNA fragment harboring TnsABC and T7 terminator was amplified from plasmid pTnsABC using primers ZZ21040/177; the DNA fragment containing *oriT*, *trfA*, *oriV* and *tetR* was amplified from plasmid pCas12a-λRed using primers ZZ21075/173 and ZZ21075/174. pQCascade-TnsABC was generated by fusing these three DNA fragments by Gibson assembly. In this plasmid, QCascade-TnsABC was expressed under the constitutive promoter P_J23119_.

Construction of the pDonor(RL-M)-crRNA-CmR plasmid series derived from pCrRNA-V [21,27]. The DNA fragment harboring two MmeI restriction enzyme cutting sites at both ends for Tn-seq, right end (RE), cargo sequence CmR, and left end (LE) was amplified from pDonor using primers ZZ21044/045 and digested by EcoRI/NdeI; the DNA fragment carrying the crRNA expression cassette with the spacer sequence replaced by two reversed *Bsa*I sites was annealed from ZZ21046/047, ZZ21048/049, ZZ21050/051. The two *Bsa*I sites enabled one-step insertion of the crRNA spacer using the Golden Gate cloning method (see below). Then, the two fragment, along with EcoRI/NdeI-digested pCrRNA-V, were ligated by T4 DNA ligase to yield plasmid pDonor(RL-M)-crRNA-CmR. In this plasmid, the crRNA was expressed as a processed repeat-spacer-repeat unit (a 32-nt spacer flanked by two 28-nt arrays) through the constitutive promoter P_J23119_. Subsequently, a series of plasmids (pDonor(RL-M)-crRNA-CmR-X) for testing the transposon insertion system were constructed by inserting a 32-bp double-stranded overhang spacer between its two *Bsa*I sites via Golden Gate cloning. The spacers were generated by annealing and phosphorylation with two primers with partial reverse complement sequences (Table S3). The spacer “5′-GTTGTCTGACACTTGTCACAAACCGCTAGGAG-3′” on pDonor(RL-M)-crRNA-CmR-NC did not target any sequence on the PAO1 or phage S1 genome.

The gene *acrVA1* driven by Ptac [29] was synthesized by Sangon Biotechnology (Shanghai, China) and amplified by primers ZZ21159/160. The fragment, along with pDonor(RL-M)-crRNA-CmR, was digested by BamHI and XbaI, and ligated by T4 DNA ligase to yield plasmid pDonor(RL-M)-crRNA-*acrVA1*. Others operations were conducted as described above.

### 2.3. Design of crRNA Spacers

crRNAs were designed using the INTEGRATE guide RNA design tool via GitHub (https://github.com/sternberglab/INTEGRATE-guide-RNA-tool (accessed on 13 May 2021)) [10,21]. Spacers were selected based on the following criteria: (1) insertion sites located in the first half of the open-reading frame (*orfs*) to ensure loss of expression, (2) GC content between 30–70%, (3) ensuring that the designed crRNAs did not share similar sequences with the *P. aeruginosa* PAO1 genome (Table S3).

### 2.4. Preparation P. aeruginosa PAO1 Cells for Transposon Insertion

The plasmids pQCascade-TnsABC and pDonor-crRNA series were separately transferred into *P. aeruginosa* PAO1 by electroporation, as previously described [27]. After pulsing, cells were recovered in 1 mL antibiotic-free LB liquid medium and incubated at 37 °C for 1.5 h. Subsequently, they were plated and cultured at 37 °C for 24~48 h on LB agar plates supplemented with tetracycline 100 μg/mL and gentamycin 50 μg/mL.

For transposon insertion experiments, transformants were identified by colony PCR. The correct transformants were then selected and plated on LB agar plates with tetracycline (100 μg/mL) and gentamycin (50 μg/mL) for a second time round of enrichment instead of growing in liquid culture. Alternatively, after being plated at 37 °C for 24~48 h on LB agar plates supplemented with tetracycline and gentamycin as above. All the recovered cells were scraped from the plates using a spreader and suspended in 1 mL of LB (Ca^2+^) with tetracycline (100 μg/mL) and gentamycin (50 μg/mL).

### 2.5. Transposon Insertion Experiments

Inserted phages were obtained by transposition plaque assay. A mixture of 0.1 mL of phage S1 with suitable dilutions (10^6^ pfu/mL, 10^5^ pfu/mL, and 10^4^ pfu/mL) and 0.3 mL of enriched cells was prepared. This mixture was then mixed with 5 mL of pre-warm top agar (0.6%, 50 °C) and poured onto LB agar. After drying at room temperature for about 10 min, the plates were incubated at 37 °C for approximately 20 h. Antibiotics were added to both the top and bottom agar during this step.

### 2.6. PCR and Sequencing Analysis of Transposon Insertion Products

After the transposon insertion experiments, the top and base agar of the double-layer agar plate was divided and transferred into 50 mL tubes containing 30 mL of SM buffer (100 nM NaCl, 8 mM MgSO_4_, and 50 mM Tris-HCl, pH 7.5) plus 300 μL of chloroform. After a 3 h incubation at room temperature, centrifugation and filtration (0.2 μm filter) removed agar and host cell debris to obtain a phage–lysate mix. For the enriched inserted phages, well-isolated plaques were transferred into 1.5 mL Eppedoff tubes containing 200 μL of SM buffer and 2 μL of chloroform. They were then incubated at room temperature for 1 h with mixing every few minutes.

A portion of the aliquots was denatured at 95 °C for 10 min, and 1.6 μL of the sample was used as template for PCR using KOD FX DNA polymerase [30]. The amplified DNA was verified by agarose gel electrophoresis and then sent for Sanger sequencing (Sangon Biotech (Shanghai, China) Co., Ltd.). Primer pairs contained one genome-specific primer and one transposon-specific primer, detecting both upstream and downstream of the target site for all possible insertion orientations.

### 2.7. Extraction of Phage Genomes

Genomic DNA was extracted as previous described with some modifications [25,31,32]. In brief, phage propagation was initially performed. A 100 mL of PAO1 culture (OD_600_ of 0.4–0.6) was infected with 100 μL of phage stocks (10^10^–10^11^ pfu/mL) and grown at 37 °C for lysis (4–5 h). Subsequently, 2 mL of chloroform was added, and the cells were incubated with shaking for 15 min at 37 °C to obtain the lysates. After centrifugation (8000× *g*, 10 min), the supernatant was transferred to new tubes. The genomic DNA and RNA of the host was removed by treating with 10 µg/mL DNase I and RNase for 1 h at 37 °C. The lysate was then supplemented with 10% (*w*/*v*) PEG 8000 and stored at 4 °C overnight to precipitate the phage particles.

After centrifugation (10,000× *g*, 30 min), the pellets were suspended in 600 μL of SM buffer, and 200 μL of chloroform was added to extract the PEG and the bacterial debris. Centrifuge at 8000× *g* for 5 min was performed, followed by treatment with 10 µg/mL DNase I and RNase for 1 h at 37 °C, and then incubation at 65 °C for 20 min to inactivate enzymes. The liquid was then added with 30 µL of 10% (*w*/*v*) SDS and 3 µL of 20 mg/mL proteinase K. The mixture was incubated for 20 min at 56 °C. Subsequently, 100 µL of 5 M NaCl and 80 µL of CTAB (cetyltrimethylammonium bromide)/NaCl solution were added and incubated for 10 min at 65 °C. Next, the mixture was sequentially treated with one volume of chloroform, one volume of phenol/chloroform/isoamyl alcohol (25:24:1), and one volume of chloroform, to gradually purify the genomic DNA. The aqueous phase was collected, and 0.7 volumes of isopropanol were added to precipitate the DNA. After centrifugation at 13,000× *g* for 15 min at 4 °C, the purified DNA pellet was washed thrice with 500 µL of ice-cold 70% ethanol and left to dry. The air-dried pellets were suspended in 100 µL ddH_2_O. The DNA concentration was measured using a Nanodrop2000 (Thermo Fisher Scientific, Waltham, MA, USA). The purified DNA was used for Tn-seq by Woosen Biotechnology (Hangzhou, China).

## 3. Results

### 3.1. Establishment of a Two-Plasmid Transposon Insertion System for P. aeruginosa Phage S1

**Figure 1:**
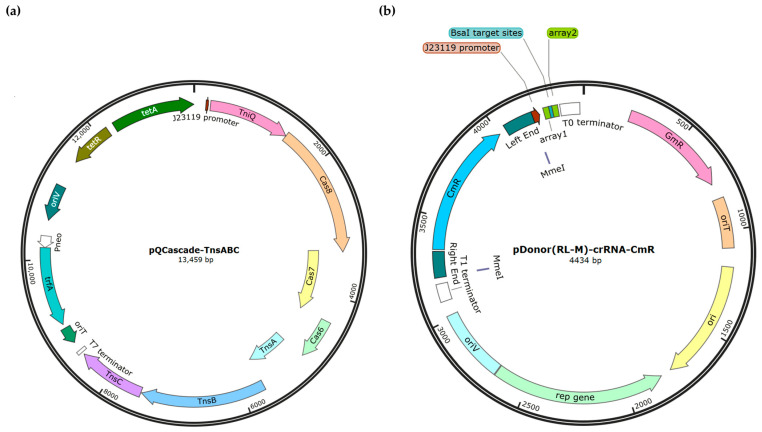
Maps of pQCascade-TnsABC and pDonor(RL-M)-crRNA-CmR plasmids created with SnapGene 4.1 (GSL Biotech LLC). (**a**) Plasmid pQCascade-TnsABC cintains the *tniQ-cas8-cas7-cas6* operon (QCascade) and the *tnsA-tnsB-tnsC* operon (TnsABC), expressed under the control of the constitutive P_J23119_ promoter. The tetracycline-resistance marker genes *tetA* and *tetR* are present for resistance in *E. coli* and *P. aeruginosa*; *oriV*, origin of replication of the broad-host-range plasmid RK2; *trfA*, plasmid replication initiator protein; *oriT*, origin of transfer for conjugation. (**b**) Plasmid pDonor(RL-M)-crRNA-CmR comprises the right end (RE), a cargo sequence (chloramphenicol resistance gene CmR), the left end (LE), two MmeI cutting sites at both ends, and the crRNA cassette transcribed through the J23119 promoter; *Bsa*I, recognition sites of type IIS restriction endonuclease for the insertion of the 32-nt spacer in the crRNA; the crRNA was expressed as a processed repeat-spacer-repeat unit, spacer flanked by two 28-nt arrays; GmR gene, the gentamycin-resistance gene.

In our initial efforts, we aimed to investigate the transposon insertion system for identifying non-essential genes within the phage S1 genome. To facilitate plasmid transformation and crRNA library construction, we streamlined the three-plasmid system (pQCascade, pTnsABC, and pDonor) into a two-plasmid system, pQCascade-TnsABC and pDonor(RL-M)-crRNA-CmR-X (referred to as pcrRNA-CmR-X), where X represents the specific target genes. Specifically, the plasmid pQCascade-TnsABC expressed the *tniQ-cas8-cas7-cas6* operon (QCascade) and the *tnsA-tnsB-tnsC* operon (TnsABC) under the control of a constitutive promoter P_J23119_ (Figure 1a). pcrRNA-CmR-X contained the right end (RE), a cargo sequence (chloramphenicol resistance gene CmR), the left end (LE), two MmeI cutting sites at both ends to facilitate Tn-seq, and the crRNA. The crRNA was expressed as a repeat-spacer-repeat unit, consisting of a 32-nt spacer flanked by two 28-nt arrays, also driven by P_J23119_ (Figure 1b).

**Figure 2:**
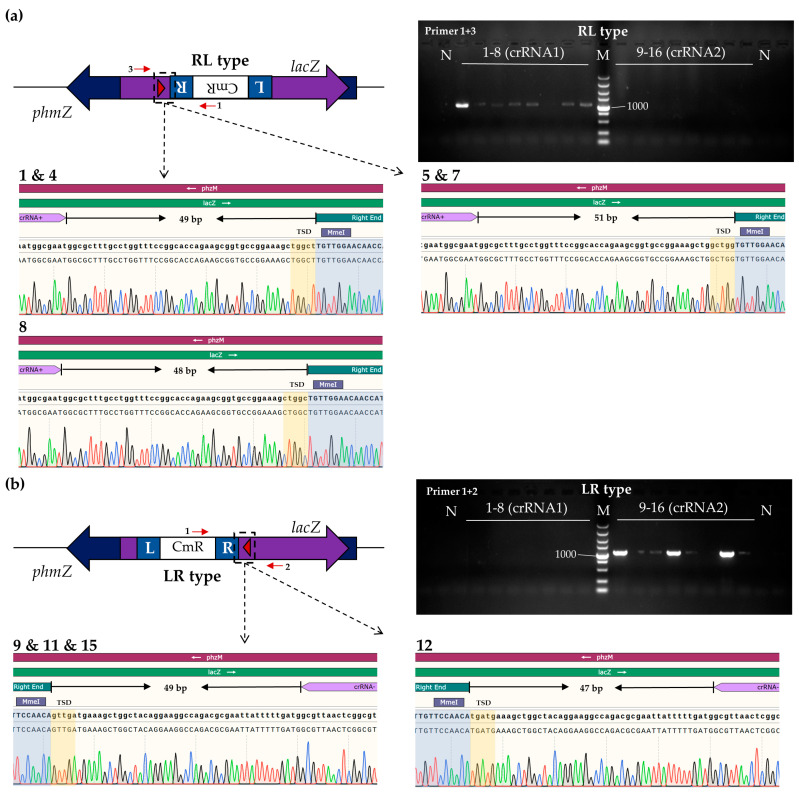
Analysis of *P. aeruginosa* strain isolates containing *lacZ*-inserted transposons. (**a**) Upper left panel, schematic diagram of RL type insertion by the two-plasmid system; upper right panel, colony PCR for the insertion in *lacZ*; lower panel, Sanger sequencing results. (**b**) Upper left panel, schematic diagram of LR type insertion; upper right panel, colony PCR for the insertion in *lacZ*; lower panel, Sanger sequencing results. The red arrows indicate the primers used in colony PCR for verifying the transposon insertion. The red triangle represents the spacer. L, left transposon end; R, right transposon end. M, Takara 5000 bp DNA ladder; 1–8, randomly selected colonies grown on plate after transformation with plasmid pQCascade-TnsABC and pcrRNA-CmR-*lacZ*-1; 9–16, randomly selected colonies grown on plate after transformation with plasmid pQCascade-TnsABC and pcrRNA-CmR-*lacZ*-2; N, blue colony; TSD, target-site duplication.

The functionality of the two-plasmid system was assessed in the *P. aeruginosa* strain PAO1 *phzM::lacZ* via blue-white selection [27]. Three crRNA plasmids, pcrRNA-CmR-*lacZ*-1/2, targeting *lacZ* genomic sites on the opposite strand, and the negative control plasmid pcrRNA-CmR-NC with no target site in the PAO1 or S1 genome, were constructed (Table S2). After separately introducing the plasmids pQCascade-TnsABC and pcrRNA-CmR into PAO1 cells through electroporation, cells were plated on X-gal plates at specific dilutions for 48 h, resulting in white and blue colonies (Figure S1a). All colonies on the negative control plate appeared blue (Figure S1b). White colonies on the pcrRNA-CmR-*lacZ*-1/2 plates suggested transposition occurring on *lacZ* guided by these two crRNAs, indicating that the two-plasmid transposon insertion system might function for PAO1. PCR was performed using a genome-specific primer and a transposon-specific primer, and only white colonies were found to product PCR fragments. Sanger sequencing confirmed transposition in white colonies, with integration events occurring 49, 51, and 48 bp from the 3′ edge of the target site for pcrRNA-CmR-*lacZ*-1 (Figure 2a), and 49 and 47 bp for pcrRNA-CmR-*lacZ*-2 (Figure 2b), consistent with the three-plasmid system [21]. The transposition efficiencies were approximately 8.5% for both pcrRNA-CmR-*lacZ*-1 (10/117) and pcrRNA-CmR-*lacZ*-2 (14/165) in insertion of PAO1 *lacZ* gene (Table S1). These results demonstrated the success of the two-plasmid transposon insertion system for gene insertion in *P. aeruginosa* strain PAO1.

We then investigated the applicability of the system for phage S1. We first targeted the non-essential gene *orf073*. We constructed four plasmids, pcrRNA-CmR-*orf073*-1~4, with half of the crRNAs located on the opposite strand. These crRNA plasmids were separately electroporated into PAO1 electrocompetent cells harboring pQCascade-TnsABC. Surprisingly, co-introduction of both plasmids resulted in cells being able to survive only on solid medium and not in liquid culture for unknown reasons. Consequently, solid medium was selected to support cell survival.

**Figure 3:**
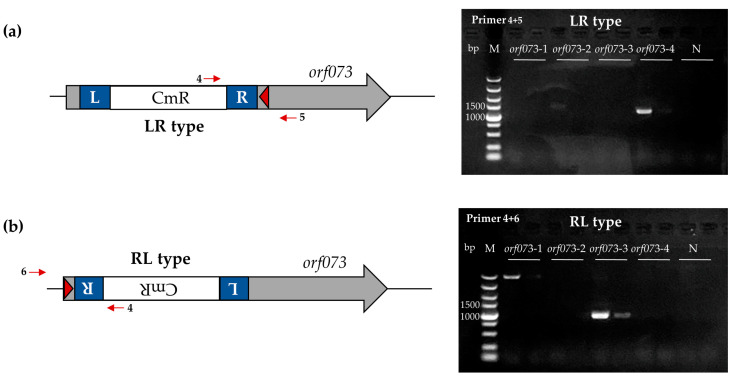
CRISPR RNA-guided transposon insertion system could function for phage S1. (**a**) Left panel, schematic diagram of LR type phage S1 *orf073* CmR insertion by the two-plasmid transposon insertion system; right panel, PCR results of phage lysate for the insertion in *orf073*. (**b**) Left panel, schematic diagram of RL type insertion; right panel, PCR results of phage lysate for the insertion in *orf073*. The red arrows indicate the primers used in PCR for verifying the transposon insertion. The red triangle represents the spacer. L, left transposon end; R, right transposon end. M, Takara 5000 bp DNA ladder; *orf073*-1~4, phage lysate mix obtained after the transposon insertion experiment with plasmids pQCascade-TnsABC and pcrRNA-CmR-*orf073*-1~4; N, negative control.

PCR of the phage lysate mix was used to determine the occurrence of transposon insertion in phage S1. PCR products (with a genome-specific primer and a transposon-specific primer, as described above) were observed in experiments with pcrRNA-CmR-*orf073*-3/4 but not with pcrRNA-CmR-NC or pcrRNA-CmR-*orf073*-1/2 (Figure 3), indicating that the two-plasmid transposon insertion system can be employed to insert target fragments into phage S1.

Although the two-plasmid system demonstrated the ability to catalyze insertion in phage S1, its efficiency was notably lower (<1%). It was reported that by eliminating the left end of MmeI cutting sites from the pDonor(RL-M)-crRNA-CmR-X could improve efficiency compared to RL m [21], but for our two-plasmid system, this modification failed to improve the efficiency (Figure S2).

### 3.2. Enrichment of Inserted Phages by AcrVA1

**Figure 4:**
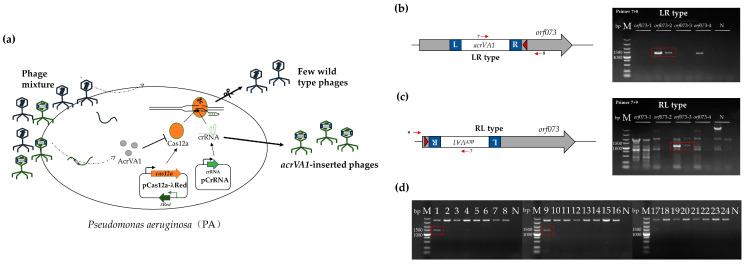
AcrVA1 can work as a selection marker for enriching integrated phages. (**a**) Schematic diagram illustrating the enrichment of inserted phages by AcrVA1. *acrVA1*-inserted phages can express AcrVA1 proteins, protecting them from interference. (**b**) Left panel, schematic diagram of LR type *acrVA1* insertion; right panel, PCR results of the insertion in *orf073*. (**c**) Left panel, schematic diagram of RL type *acrVA1* insertion; right panel, PCR results of the insertion. (**d**) PCR results of the enrichment of *acrVA1*-inserted phages. The red arrows indicate the primers used in PCR for verifying the transposon insertion. The red triangle represents the spacer. L, left transposon end; R, right transposon end. M, Takara 5000 bp DNA ladder; *orf073*-1~4, phage lysate mix obtained after the transposon insertion experiment with plasmids pQCascade-TnsABC and pcrRNA-*acrVA1*-*orf073*-1~4; 1–24, randomly selected plaques grown on plate after enrichment; N, negative control.

Unable to enhance the efficiency of the two-plasmid system by altering the number of MmeI sites, we sought a different method to enrich the inserted phages. Currently, there are limited selection makers available for phage engineering, and marker genes essential for phage propagation are scarce, found only in a few model phages [33,34,35]. Inspired by the editing approach of the *Sulfolobus islandicus* rod-shaped virus 2 (SIRV2), which uses the anti-CRISPR protein AcrID1 (SIRV3 gp02) to overcome type I-D CRISPR-Cas immunity [36], we chose AcrVA1, a broad-spectrum inhibitor of the commonly used Cas12a with modest inhibition [29], as the transposon cargo sequence for enriching inserted phages (Figure 4a). Given our establishment of CRISPR-Cas12a as an editing system for phage S1 (unpublished), this choice ensures that only phages carrying the inserted *acrVA1* gene can evade CRISPR-Cas12a interference, enabling their survival, accumulation, and dominance in the enrichment field.

To this end, we constructed five crRNA plasmids, namely pcrRNA-*acrVA1*-NC and pcrRNA-*acrVA1*-*orf073*-1~4, replacing the cargo sequence CmR of pcrRNA-CmR-NC and pcrRNA-CmR-*orf073*-1~4 with Ptac-*acrVA1*. Subsequently, we cultured the phages on solid media and conducted plaque assays to assess the successful insertion of *acrVA1*. In experiments utilizing pQCascade-TnsABC and pcrRNA-*acrVA1*-*orf073*-2/3, PCR products (with a genome-specific primer and a transposon-specific primer, as described above) within the phage-lysate mixture were detected for *acrVA1* insertion (Figure 4b,c).

To gauge the efficiency of AcrVA1, we needed to identify a high-activity crRNA (EOP < 0.1) capable of targeting the majority wild-type phage S1 in the presence of the Cas12a protein. pCrRNA(Cas12a) were one of the low EOP crRNAs we had already tested, so we selected this crRNA to enrich inserted phages (Table S2). Following interference by the CRISPR-Cas12a system for the inserted phages in experiment using pcrRNA-*acrVA1-orf073*-2, we randomly selected 24 plaques. Among them, 2 out of 24 displayed the expected PCR products (Figure 4d). Therefore, the efficiency of AcrVA1 enrichment was 8.33%, representing an improvement of more than 10 times compared to the non-selection-marker. In summary, these results indicated the success in using AcrVA1 as a selection maker to enrich inserted phages

### 3.3. Transposon Insertions of Phage S1

We next evaluated the capability of our two-plasmid transposon insertion system in identifying non-essential genes of the phage S1. Ten genes, including *orf026* (putative major structural protein) and *orf058* (putative DNA polymerase III alpha subunit), as well as *orf010*, *orf014*, *orf043*, *orf055*, *orf072*, *orf075*, *orf081*, and *orf086* (all hypothetical proteins), were selected for testing. Four crRNAs were designed for each gene, targeting the opposite strand and all located in the first half of the genes to prevent expression of a disrupted gene. crRNA plasmids, pcrRNA-*acrVA1*-X-1~4, were constructed from pcrRNA-*acrVA1*-NC, where X represents the respective gene. Individual transpositions in phage S1 produced expected PCR products from *orf010*, *orf014*, *orf072*, *orf081*, and *orf086* (Figure S3), indicating successful transposon insertions. However, *orf026*, *orf058*, *orf043*, *orf055*, and *orf075* could not be inserted, suggesting their potential indispensability.

In contrast, simultaneous transposition of the selected ten open-reading frames using forty crRNA plasmids did not yield the expected PCR products (Figure S4a). This issue persists in smaller groups of crRNA plasmids, including *orf010* + *orf014*, *orf010* + *orf014* + *orf072*, *orf010* + *orf014* + *orf072* + *orf081*, and *orf010* + *orf014* + *orf072* + *orf081* + *orf086* (Figure S4b). This challenge requires further investigation and resolution.

### 3.4. Discovering Site-Specific Integration by Tn-Seq

We utilized Tn-seq, a transposon insertion sequencing method, for accurately determining genetic interactions on a genome-wide scale [37]. More specifically, we constructed the pDonor(RL-M)-crRNA plasmid with two MmeI sites [10,21]. After transposition in phage S1, we amplified phage lysates, extracted phage genomes, and analysis of the Tn-seq data unveiled that the insertion sites of Tn-seq reads predominantly occurred in *orf010*, *orf014*, *orf072*, *orf081*, and *orf086*, accounting for approximately 99.4% of the total reads (Figure S5). This result demonstrates the exquisite specificity of our two-plasmid transposon insertion system, affirming confidence in identifying non-essential genes.

## 4. Discussion

In this work, we developed a two-plasmid CRISPR RNA-guided transposon insertion system for phage S1, utilizing the anti-CRISPR protein AcrVA1 as the transposon cargo to counteract CRISPR-Cas12a, enriching transposon inserted phages. Successful transposition of CmR into the PAO1 *phzM::lacZ* loci indicated compatibility in *P. aeruginosa* strain PAO1. Moreover, this system was applicable to phage S1. Additionally, *acrVA1* was transposed into *orf073*, *orf010*, *orf014*, *orf072*, *orf081*, and *orf086* of the phage S1 genome, affirming our confidence in identifying non-essential genes.

Our study presents the first site-specific, programmable in vivo phage transposon insertion system, holding promise for future applications across a broader range of phages and facilitating the insertion of genetic cargos. The ability to achieve site-specific DNA integration using the transposon insertion approach eliminates the need for homology arms in the donor DNA, double-strand breaks in the target DNA, and host or donor DNA repair factors [21]. This contributes a new tool for the identification of non-essential genes for phages.

Our initial design for the plasmid pDonor(RL-M)-crRNA aimed to generate large crRNA libraries, enabling high-throughput screening, like Tn-seq, of phage genes that are not easily accessible by random transposase strategies [10]. Unfortunately, due to the low efficiency of our two-plasmid system in phage S1, this purpose has not been realized. One potential contributing factor for this low efficiency may be the limited window phase of phage transposition. The two-plasmid system can only catalyze transposition in phages during the latent period, which is a short time, just a few minutes [38]. Additionally, plasmid loss is also a possible cause [39].

It is important to further improve the efficiency of our two-plasmid transposon insertion system. We attempted to replicate the reported single-plasmid system pSPIN, an advanced version of the three-plasmid system with greater efficiency [10]. However, constructing the pSPIN plasmid with a crRNA target for phage S1 failed, leading us to consider the possibility that the crRNA may have toxic effects on *P. aeruginosa* PAO1. Future studies might consider using an inducible promoter to prevent potential leaky expression of the QCascade-TnsABC complex, thereby preventing premature loss of the plasmid. The growing number of RNA-guided CRISPR-Cas transposase systems [40] suggests the potential to find a suitable and efficient transposase system for different phages. Efficient multiplexing insertions may also be achieved by employing multi-spacer CRISPR arrays in pDonor-crRNA following identical protocols as reported [10].

## Data Availability

The data presented in this study are available in the article.

## Supplementary Materials

The following supporting information can be downloaded at: https://www.mdpi.com/article/10.3390/v16030422/s1, Figure S1: Phenotypic analysis of the CmR-inserted PAO1 *phzM::lacZ* mutant; Figure S2: The efficiency of modified CRISPR RNA-guided transposon insertion system; Figure S3: Phage lysate PCR for the insertion of 10 open-reading frames; Figure S4: Simultaneous transposition of phage S1; Figure S5: Genome-wide analysis of the transposon insertion for phage S1; Table S1: Summary of the transposition efficiencies using transposon insertion system; Table S2: All the phage, bacterial strains, and plasmids used in this study; Table S3: Spacer sequences for CRISPR RNA-guided transposases; Table S4: The primers used for the construction of plasmids and colony or plaque PCR.
