# Supplementary material for: CRISPR RNA-Guided Transposases Facilitate Dispensable Gene Study in Phage
Source: Viruses. 2024 Mar 9;16(3):422. doi: 10.3390/v16030422 (PMC10974960; doi:10.3390/v16030422)
Supplement: Supplementary file 1 [file viruses-16-00422-s001.zip › viruses-2867715-supplementary.pdf]

Figures

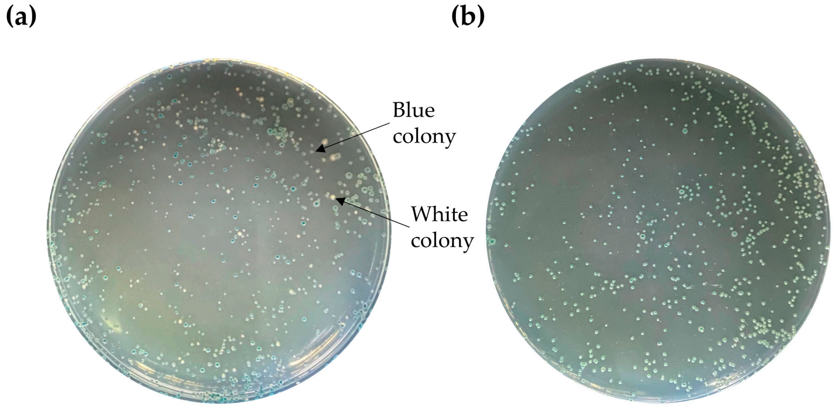

Figure S1. Phenotypic analysis of the CmR-inserted PAO1 *phzM::lacZ* mutant. (a) PAO1 cells containing pQCascade-TnsABC and pcrRNA-CmR-*lacZ*-1/2 were plated on X-gal-containing plates. White colonies indicated *lacZ*-inactivating transposon insertions. (b) PAO1 cells containing pQCascade-TnsABC and pcrRNA-CmR-NC (non-targeting) were plated on X-gal plate as negative control.

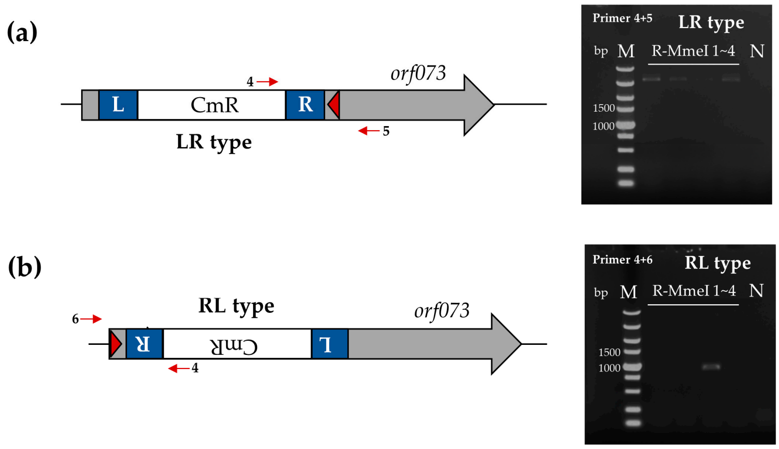

Figure S2. The efficiency of modified CRISPR RNA-guided transposon insertion system. (a) Left panel, schematic diagram of LR type CmR insertion in phage S1 *orf073* by the two-plasmid transposon insertion system; right panel, phage lysate PCR for *orf073* insertion with R-MmeI crRNA plasmids. (b) Left panel, schematic diagram of RL type CmR insertion; right panel, PCR for insertion with R-MmeI crRNA plasmids. The red arrows indicate PCR primer locations and the red triangle represents the spacer. L, left transposon end; R, right transposon end. M, Takara 5,000 bp DNA ladder; R-MmeI 1~4, phage lysate obtained after the transposon insertion experiments with plasmids pQCascade-TnsABC and pcrRNA(R-M)-CmR-*orf073*-1~4; N, negative control.

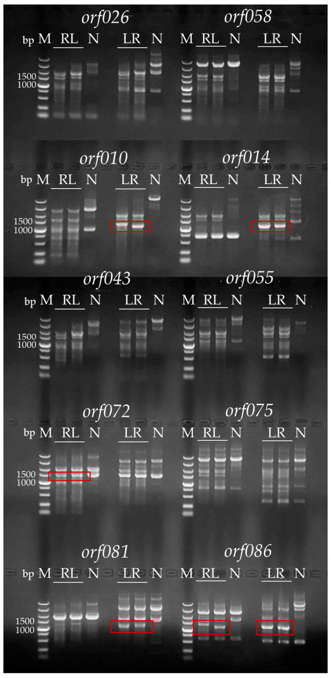

Figure S3. Phage lysate PCR for the insertion of 10 open-reading frames. M, Takara 5,000 bp DNA ladder; X, the respective gene; *orfX*, phage lysate obtained after transposon insertion with plasmids pQCascade-TnsABC and pcrRNA-*acrVA1*-*orfX*; N, negative control. Possible destination PCR fragments are marked with red boxes.

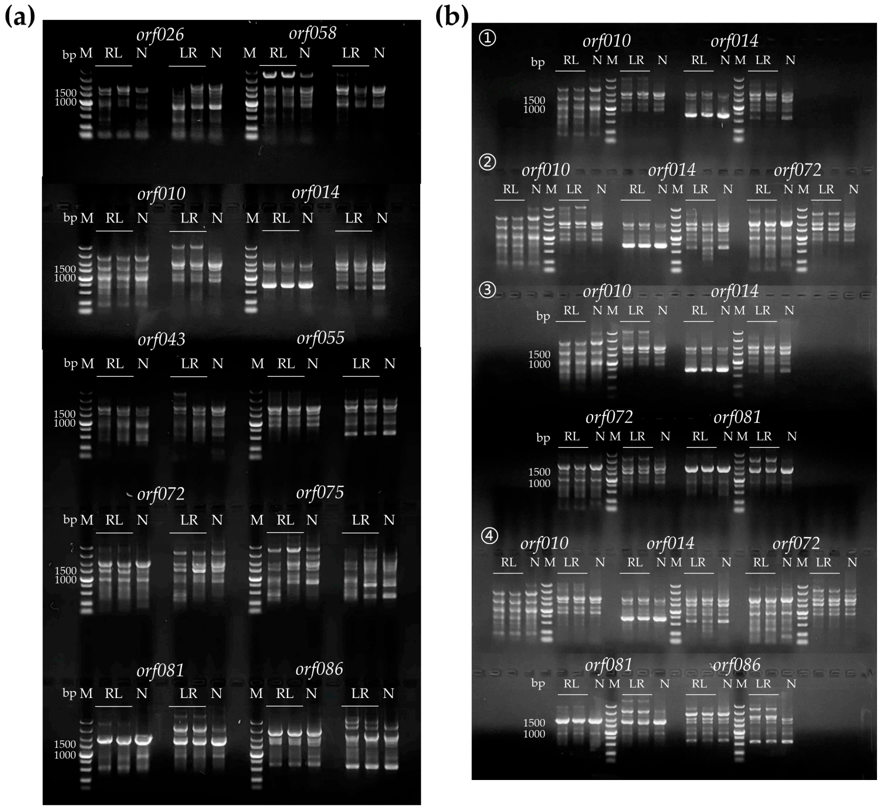

Figure S4. Simultaneous transposition of phage S1. (a) Phage lysate PCR of the simultaneous insertion of 10 open-reading frames. (b) Simultaneous transposition of phage S1

in smaller crRNA plasmid groups. ①, *orf010+orf014*; ②, *orf010+orf014+orf072*; ③, *orf010+orf014+orf072+orf081*; ④, *orf010+orf014+orf072+orf081+orf086*. RL type and LR type denote transposition products in which the right or left transposon end is on the left, respectively. M, Takara 5,000 bp DNA ladder; X, the respective gene; *orfX*, phage lysate obtained after transposon insertion with plasmids pQCascade-TnsABC and crRNA group. N, negative control.

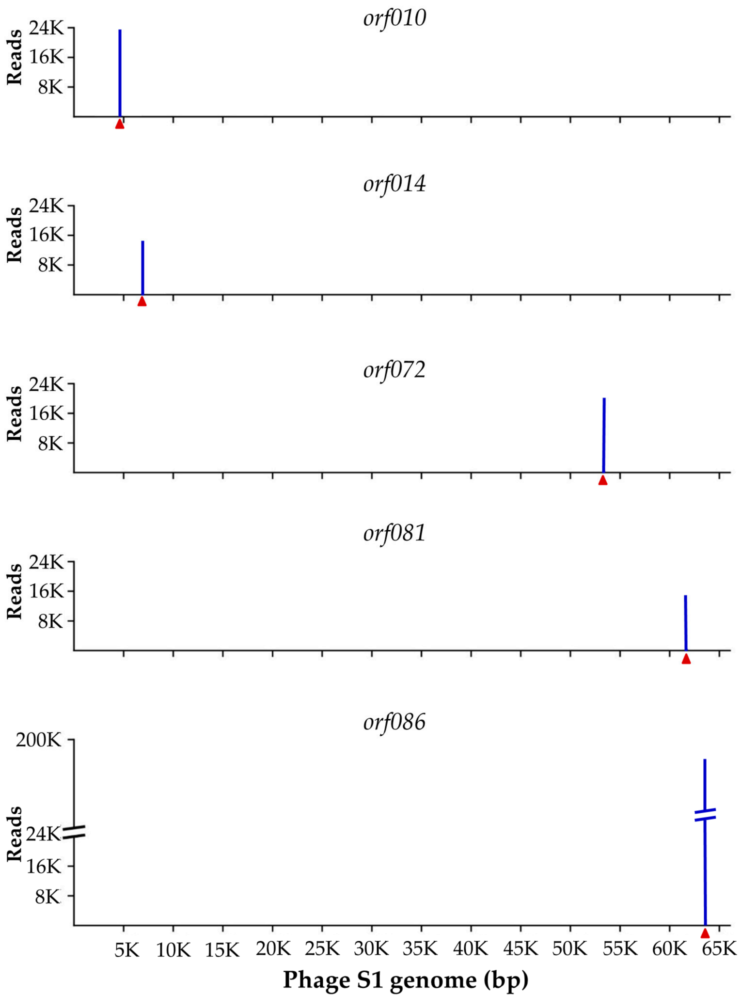

Figure S5. Genome-wide analysis of the transposon insertion for phage S1. Genome-wide distribution of genome-mapping Tn-seq reads from transposon insertion experiments with 40 crRNAs targeted phage S1 genome for the two-plasmid V. cholerae transposon. The location of each target site is denoted by a maroon triangle.

Tables

Tables S1 Summary of the transposition efficiencies using transposon insertion system.

Organism	Target gene	Transposition efficiency	Transposition efficiency after enrichment
P. aeruginosa PAO1	*phzM::lacZ*	8.5% (10/117, 14/165)	-*
P. aeruginosa phage S1	*orf073*	＜ 1%	8.33% (2/24)
	orf081	-	-
	orf010	-	-
	orf014	-	-
	orf072	-	-
	orf086	-	-

* -: not determined.

Tables S2 All the phage, bacterial strains, and plasmids used in this study

Phage, strain, or plasmid	Relevant characteristic	Source or reference
Phage		
vB_PaeM_SCUT-S1	GenBank MK340760	Lab stock [1]
Strain		
E. coli DH5*α*	F^- φ80d *lac*ZΔM15 Δ(*lac*ZYA-*arg*F) U169 *end* A1 *rec*A1 *hsd*R17 (r_K^-, m_K^+) *sup*E44λ- *thi*-1 *gyr*A96 *rel*A1 *pho*A	TransGen Biotech
P. aeruginosa PAO1	Wild type	Lab stock
P. aeruginosa PAO1 *phzM::lacZ*	PAO1 with *lacZ* insertion in gene *phzM*	Lab stock [2]
Plasmid		
pQCascade-entry (BsaI-stuffer)	CloDF13, Sm^R, P_{T7}-CRISPR array (two BsaI), P_{T7}-TniQ-Cas8-Cas7-Cas6	Addgene 130633 [3]
pTnsABC	ColA, Kan^R, P_{T7}-TnsA-TnsB-TnsC	Addgene 130634 [3]
pDonor	ColE1/pMB1/pBR322/pUC ori, Amp^R, Right end-Cm^R-Left end	Addgene 130635 [3]
pCas12a-*λ*Red	pCas-Cpf1, araC-pBAD-λRed, oriT, trfA, oriV, tetR	Lab stock [2]
pCrRNA-V	ColE1/pMB1/pBR322/pUC ori, pRO1600 ori, Gm^R, P_{J23119}-crRNA, P_{sacB}-sacB	Lab stock [2]
pQCascade-TnsABC	P_{J23119}-TniQ-Cas8-Cas7-Cas6-TnsA-TnsB-TnsC, oriT, trfA, oriV, tetR	This work
pDonor(RL-M)-crRNA-CmR	ColE1/pMB1/pBR322/pUC ori, pRO1600 ori, Gm^R, P_{J23119}-CRISPR array (two BsaI), Right end (MmeI)-Cm^R-Left end (MmeI)	This work

pDonor(RL-M)-crRNA-CmR-NC	Derived from pDonor(RL-M)-crRNA-CmR, crRNA NC was inserted	This work
pDonor(RL-M)-crRNA-CmR-*lacZ*-1/2	Derived from pDonor(RL-M)-crRNA-CmR, crRNA *lacZ*-1/2 was inserted	This work
pDonor(RL-M)-crRNA-CmR-*orf073*-1~4	Derived from pDonor(RL-M)-crRNA-CmR, crRNA *orf073*-1~4 was inserted	This work
pDonor(R-M)-crRNA-CmR-*orf073*-1~4	Derived from pDonor(RL-M)-crRNA-CmR-*orf073*-1~4, Left end MmeI sites were removed	This work
pDonor(RL-M)-crRNA-*acrVA1*-NC	Derived from pDonor(RL-M)-crRNA-CmR-NC, *acrVA1* was replaced of Cm^R	This work
pDonor(RL-M)-crRNA-*acrVA1*-*orf073*-1~4	Derived from pDonor(RL-M)-crRNA-CmR-*orf073*-1~4, *acrVA1* was replaced of Cm^R	This work
pCrRNA(Cas12a)	ColE1/pMB1/pBR322/pUC ori, pRO1600 ori, Gm^R, P_{J23119}-crRNA (CTCAAGCCCAAATCATCGACGCG), enrichment	This work
pDonor(RL-M)-crRNA-*acrVA1*	ColE1/pMB1/pBR322/pUC ori, pRO1600 ori, Gm^R, P_{J23119}-CRISPR array (two BsaI), Right end (MmeI)-*acrVA1*-Left end (MmeI), derived from pDonor(RL-M)-crRNA-CmR by changing Cm^R sequence	This work
pDonor(RL-M)-crRNA-*acrVA1*-*orf026*-1~4	Derived from pDonor(RL-M)-crRNA-*acrVA1*, crRNA *orf026*-1~4 was inserted	This work
pDonor(RL-M)-crRNA-*acrVA1*-*orf058*-1~4	Derived from pDonor(RL-M)-crRNA-AcrVA1, crRNA *orf058*-1~4 was inserted	This work
pDonor(RL-M)-crRNA-*acrVA1*-*orf010*-1~4	Derived from pDonor-crRNA-AcrVA1, crRNA *orf010*-1~4 was inserted	This work

pDonor(RL-M)-crRNA-*acrVA1*-*orf014*-1~4	Derived from pDonor-crRNA-AcrVA1, crRNA *orf014*-1~4 was inserted	This work
pDonor(RL-M)-crRNA-*acrVA1*-*orf043*-1~4	Derived from pDonor-crRNA-AcrVA1, crRNA *orf043*-1~4 was inserted	This work
pDonor(RL-M)-crRNA-*acrVA1*-*orf055*-1~4	Derived from pDonor-crRNA-AcrVA1, crRNA *orf055*-1~4 was inserted	This work
pDonor(RL-M)-crRNA-*acrVA1*-*orf072*-1~4	Derived from pDonor-crRNA-AcrVA1, crRNA *orf072*-1~4 was inserted	This work
pDonor(RL-M)-crRNA-*acrVA1*-*orf075*-1~4	Derived from pDonor-crRNA-AcrVA1, crRNA *orf075*-1~4 was inserted	This work
pDonor(RL-M)-crRNA-*acrVA1*-*orf081*-1~4	Derived from pDonor-crRNA-AcrVA1, crRNA *orf081*-1~4 was inserted	This work
pDonor(RL-M)-crRNA-*acrVA1*-*orf086*-1~4	Derived from pDonor-crRNA-AcrVA1, crRNA *orf086*-1~4 was inserted	This work

Tables S3 Spacer sequences for CRISPR RNA-guided transposases

Spacer	Sequence (5′ to 3′)	CG (%)	Direction
crRNA			
NC	GTTGTCTGACACTTGTCACAAACCGCTAGGAG	50	—
lacZ-1	GTTGCACCACAGATGAAACGCCGAGTTAACGC	53.125	fw
lacZ-2	TTCCCAACAGTTGCGCAGCCTGAATGGCGAAT	53.125	rv
orf073-1	GCGAGCTATTGCGATGGCGTGATCGGCGATGA	59.375	fw
orf073-2	AGGGCGGCGTCGCGTTCCACTTTATAACGGCA	59.375	rv
orf073-3	CAGTATTCCGGAGTTCGAGAAGATCGACGCCA	53.125	fw
orf073-4	ATAGGTTGTTGTAGCGATTGCGCGCCTCAGTC	53.125	rv
orf026-1	ATCGTCCAGCTCACGCAGTAAGGAGGCGTCAT	56.25	fw
orf026-2	CAGGGCGGCCACGGCTTCGTGGGTGACGTTCT	68.75	rv
orf026-3	GGCCGGCTCAACGATGCCCTGAACGATTTCCT	59.375	rv
orf026-4	ACACCAACATCCCGCTGACCAGCTGGAACGCC	62.5	fw
orf058-1	AATACTCGTGCGGTAGTCGAACAATTGGCGGA	50	rv
orf058-2	GTCTCCATAGCGCCGTGCGCGGCGACCATCGA	68.75	fw
orf058-3	ATCATCGACGATGGCGACGAATGGGGATTCGA	53.125	fw
orf058-4	GCATGATGTCTTTCCCTCAGCTCCGCGTTCGC	59.375	rv
orf010-1	CAGCGCTTGGTAGTGCTTCCGTCGCGGGCGCT	68.75	fw
orf010-2	GAGCGTGTGAAGAAGTTGAAGGACAAATATGA	40.625	rv
orf010-3	TTCAACTTCTTCACACGCTCGGCTGCGATGGC	56.25	fw
orf010-4	CTTCAGACAAAAGGAACCGATCATGACCGACC	50	rv
orf014-1	TGCGCGGCCACTTTCTCGCGATACTTGGCGAG	62.5	fw
orf014-2	TTAGCACGGCTCTTGGCGCGCTGCTCCGGGCT	68.75	fw

orf014-3	TGGAAGACGTGTCCATGGGTGCCCGCCCGAAC	65.625	rv
orf014-4	AAACAGGAGAATCGACATGAACGCAGCAGAGA	46.875	rv
orf043-1	CAGCAGCGTAATGGCCAGGGAAATCAAATCGT	50	fw
orf043-2	AGCACGTCGGTCCCTTTGAGCAATTCTTCCGG	56.25	rv
orf043-3	TTGACCGCTGCGAACATAAATCTTTATGTCAT	37.5	fw
orf043-4	AGTTCAGACAAAAGCGCAAGCACTTGTACGGG	50	rv
orf055-1	GCCAGAGTAGTGGTCTTTTGACCAAGCTCCTC	53.125	fw
orf055-2	TGGTGGACAAGGCTTCGCAGCTGGACGTCGAA	59.375	rv
orf055-3	AGGTTCAGCAGTCGATGGCCGATGTCGGTGGC	62.5	fw
orf055-4	CGGAATACCTGAAAGAGAACGCACCGGGCGAA	56.25	rv
orf072-1	CTCCAAAACAGAGGATACACTAAATGCAAGAA	37.5	fw
orf072-2	CATCCGGCCTTGGCCAAGTCGGACGCTTCGGC	68.75	rv
orf072-3	CATCTTCCGGAACTCCGGTAACGCGAACGACG	59.375	rv
orf072-4	TCTATGGATCGAAAGCCTTCCCGCGCCATTCC	56.25	fw
orf075-1	ACGACGCAAGGGGCGCCCGGAGCGACTTGCAT	68.75	fw
orf075-2	GGCGTTGAGCTTGCGGCCGTTCTCATCGGTCT	62.5	rv
orf075-3	AACGCCTACGCATGTATCTCGTCGTCAATCAA	46.875	fw
orf075-4	AGTATCGCATCTGGCCGGTCTTCGGGTTCGGC	62.5	rv
orf081-1	GTCTTGTCGCCGCCCGGCTCTGCCGGATCGAA	68.75	fw
orf081-2	GGGTCCGTGGCTGATGATCTCGGCAGGAATAT	56.25	fw
orf081-3	GAAGAGTCTACTGGCCAGATCGTGGTAAGCCC	56.25	rv
orf081-4	TTATTGAGGAGCGCACCATGCTGTACATCTGG	50	rv
orf086-1	AACATGGGTCGCAGAGCAGCTTCCGGGTCAAC	59.375	fw
orf086-2	TGGGGAACCTGGAAGGCGTAACAATCGGGAAT	53.125	rv

orf086-3	AGGAGAACGTGGAGTTTGCTGACGTATTCCTC	50	fw
orf086-4	TTCAAAAACCGAGGAACCGCCATGAAACTCTA	43.75	rv

Tables S4 The primers used for the construction of plasmids and colony or plaque PCR

Primer	Sequence (5′-3′)	Function
ZZ21038	cctaggtataatgctagcGGATCCGAATTCGAGCGAAGGAGATATACATATGTTTTTGCAAAGACCTAAACCTTA	pQCascade-TnsABC
ZZ21039	CTTGTCGCCATGGTATATCTCCTTATTAAAGGTCAGATTAAGGGTACAGGCT	
ZZ21040	AAGGAGATATACCATGGCGACAAGTTTACCTAC	
ZZ21177	GCAGCAGCCTAGGTTATTCGAAAAAGTTTTTATTTCTACCTGGTTTACTCACTCTCGGG	
ZZ21075	CTTTTTCGAATAATTCGCCATGCCCCATGGGTATGGACAGTTTT	
ZZ21173	GGGTCTTGAGGGGTTTTTTGCTGAAACCTCAGGCAttcgccatgccccatgggtatgga	
ZZ21174	CTGAGCAATAACTAGCATAACCCCTTGGGGCCTCTAAACGGGTCTTGAGGGGTTTTTTG	
ZZ21183	CTGAACTTAAGAGAGCATTTCGTCCAC	Colony PCR of pQCascade-TnsABC
ZZ21184	GAAAGCTGTAAGTGATGGAATCCCC	
ZZ21044	ccggaattcTGTTGGAACAACCATAAAATGATAA	pDonor(RL-M)-crRNA-CmR
ZZ21045	GGAATTCCATATGTGTTGGAGCAACCATAAAG	
ZZ21046	TATGttgacagctagctcagtcctaggtataatgcta	
ZZ21047	ACgctagcattatacctaggactgagctagctgtcaaCA	
ZZ21048	gcGTCGACGTGGAGATATACCATGGGTGAACTGCCGAGTAGGTAGCTGATA	
ZZ21049	TCGTTATCAGCTACCTACTCGGCAGTTCACCCATGGTATATCTCCACGTCG	

ZZ21050	ACGAGACCTCTGGTCTCGTGAACTGCCGAGTAGGTAGCTGATAACa	
ZZ21051	ctagtGTTATCAGCTACCTACTCGGCAGTTCACGAGACCAGAGGTC	
ZZ21141	gcaatccccgttttaccagtccc	Colony PCR of pDonor-crRNA
ZZ21142	AACCGAACAGGCTTATGTCAAATTTAAATCGT	
ZZ21163	tttacactttatgcttccggctcgtatg	PCR identification of *lacZ* insertion
ZZ21164	ATTCTTGCCCGCCTGATGAATG	
ZZ21165	TCGCAGTACTGTTGTAATTCATTAAGCATTCT	
ZZ21185	GGCCGGATGGGTCAATTCACA	PCR identification of *orf073* insertion
ZZ21186	GAGAAGGCCGACTCGCTCAG	
ZZ21187	GTCTATGACGAGCTGTACACGGAG	
ZZ21188	CTGAACGGTCTGGTTATAGGTACATTGAG	
ZZ21189	TGTCGGCAGAATGCTTAATGAATTACAACA	
026+F	AGTTACCTGAAGGGGGAGTAACCC	PCR identification of 10 open reading frame insertion
058+F	GAGCGGGTCAACCTCGGTAATG	
010+F	TTTTTCGGCCTCGACCGACCT	
014+F	GGCCGTGCAGAGTGATCTTGT	
043+F	ACGAACTGATGATCAGAGTATCGGTT	
055+F	TCATCGACCTGGGGAGACATCT	
072+F	CGGAATTGATCGCGTCGATCTGC	
075+F	GCCGTATGAATTTCTTACTCCCCTCTACAC	
081+F	CAGCGACGCTCAGCAGTCTTG	
086+F	AGTCGGTGAAGAGATTATGCCTGA	
026-R	TCCACTTCTTCGACGAAGAGGACCA	
058-R	CAGCCGGCCTTTCGACATAAGA	
010-R	TCATGCGGAAAGGCAGTGTTCATAAAT	
014-R	ACTATACCCATGGTTACGTCGGTTAAGG	

043-R	TAAATCCAGCCGATATCCCCCTG	
055-R	CAAGTTGCATCCGGCTATATCAACG	
072-R	AATCGTCTCCAGAGCCTCATAAAGTT	
075-R	CAATTGTTCGACGATATATGGCAGCACTAC	
081-R	TGTCCATCCTTCACAAACGTTAATCCA	
086-R	AAAATCATCGGCCTGAGAACTCTGT	
NC-F	ATAACGTTGTCTGACACTTGTCACAAACCGCTAGGAGG	
NC-R	TTCACCTCCTAGCGGTTTGTGACAAGTGTCAGACAACG	
ZZ073-1-F	ATAACGCGAGCTATTGCGATGGCGTGATCGGCGATGAG	
ZZ073-1-R	TTCACTCATCGCCGATCACGCCATCGCAATAGCTCGCG	
ZZ073-2-F	ATAACAGGGCGGCGTCGCGTTCCACTTTATAACGGCAG	
ZZ073-2-R	TTCACTGCCGTTATAAAGTGGAACGCGACGCCGCCCTG	
ZZ073-3-F	ATAACCAGTATTCCGGAGTTCGAGAAGATCGACGCCAG	
ZZ073-3-R	TTCACTGGCGTCGATCTTCTCGAACTCCGGAATACTGG	crRNA anneal primers
ZZ073-4-F	ATAACATAGGTTGTTGTAGCGATTGCGCGCCTCAGTCG	
ZZ073-4-R	TTCACGACTGAGGCGCGCAATCGCTACAACAACCTATG	
ZZ026-1-F	ATAACATCGTCCAGCTCACGCAGTAAGGAGGCGTCATG	
ZZ026-1-R	TTCACATGACGCCTCCTTACTGCGTGAGCTGGACGATG	
ZZ026-2-F	ATAACCAGGGCGGCCACGGCTTCGTGGGTGACGTTCTG	
ZZ026-2-R	TTCACAGAACGTCACCCACGAAGCCGTGGCCGCCCTGG	

ZZ026-3-F	ATAACGGCCGGCTCAACGATGCCCT GAACGATTTCCTG
ZZ026-3-R	TTCACAGGAAATCGTTCAGGGCATC GTTGAGCCGGCCG
ZZ026-4-F	ATAACACACCAACATCCCGCTGACC AGCTGGAACGCCG
ZZ026-4-R	TTCACGGCGTTCCAGCTGGTCAGCG GGATGTTGGTGTG
ZZ058-1-F	ATAACAATACTCGTGCGGTAGTCGA ACAATTGGCGGAG
ZZ058-1-R	TTCACTCCGCCAATTGTTCGACTACC GCACGAGTATTG
ZZ058-2-F	ATAACGTCTCCATAGCGCCGTGCGC GGCGACCATCGAG
ZZ058-2-R	TTCACTCGATGGTCGCCGCGCACGG CGCTATGGAGACG
ZZ058-3-F	ATAACATCATCGACGATGGCGACGA ATGGGGATTCGAG
ZZ058-3-R	TTCACTCGAATCCCCATTCGTCGCC ATCGTCGATGATG
ZZ058-4-F	ATAACGCATGATGTCTTTCCCTCAGC TCCGCGTTCGCG
ZZ058-4-R	TTCACGCGAACGCGGAGCTGAGGG AAAGACATCATGCG
ZZ010-1-F	ATAACCAGCGCTTGGTAGTGCTTCC GTCGCGGGCGCTG
ZZ010-1-R	TTCACAGCGCCCGCGACGGAAGCA CTACCAAGCGCTGG
ZZ010-2-F	ATAACGAGCGTGTGAAGAAGTTGA AGGACAAATATGAG
ZZ010-2-R	TTCACTCATATTTGTCCTTCAACTTC TTCACACGCTCG
ZZ010-3-F	ATAACTTCAACTTCTTCACACGCTC GGCTGCGATGGCG
ZZ010-3-R	TTCACGCCATCGCAGCCGAGCGTGT GAAGAAGTTGAAG
ZZ010-4-F	ATAACCTTCAGACAAAAGGAACCG ATCATGACCGACCG

ZZ010-4-R	TTCACGGTCGGTCATGATCGGTTCCT TTTGTCTGAAGG
ZZ014-1-F	ATAACTGCGCGGCCACTTTCTCGCG ATACTTGGCGAGG
ZZ014-1-R	TTCACCTCGCCAAGTATCGCGAGAA AGTGGCCGCGCAG
ZZ014-2-F	ATAACTTAGCACGGCTCTTGGCGCG CTGCTCCGGGCTG
ZZ014-2-R	TTCACAGCCCGGAGCAGCGCGCCA AGAGCCGTGCTAAG
ZZ014-3-F	ATAACTGGAAGACGTGTCCATGGGT GCCCGCCCGAACG
ZZ014-3-R	TTCACGTTCGGGCGGGCACCCATGG ACACGTCTTCCAG
ZZ014-4-F	ATAACAAACAGGAGAATCGACATG AACGCAGCAGAGAG
ZZ014-4-R	TTCACTCTCTGCTGCGTTCATGTCGA TTCTCCTGTTTG
ZZ043-1-F	ATAACCAGCAGCGTAATGGCCAGG GAAATCAAATCGTG
ZZ043-1-R	TTCACACGATTTGATTTCCCTGGCCA TTACGCTGCTGG
ZZ043-2-F	ATAACAGCACGTCGGTCCCTTTGAG CAATTCTTCCGGG
ZZ043-2-R	TTCACCCGGAAGAATTGCTCAAAGG GACCGACGTGCTG
ZZ043-3-F	ATAACTTGACCGCTGCGAACATAAA TCTTTATGTCATG
ZZ043-3-R	TTCACATGACATAAAGATTTATGTTC GCAGCGGTCAAG
ZZ043-4-F	ATAACAGTTCAGACAAAAGCGCAA GCACTTGTACGGGG
ZZ043-4-R	TTCACCCCGTACAAGTGCTTGCGCT TTTGTCTGAACTG
ZZ055-1-F	ATAACGCCAGAGTAGTGGTCTTTTG ACCAAGCTCCTCG
ZZ055-1-R	TTCACGAGGAGCTTGGTCAAAAGA CCACTACTCTGGCG

ZZ055-2-F	ATAACTGGTGGACAAGGCTTCGCAG CTGGACGTCGAAG
ZZ055-2-R	TTCACTTCGACGTCCAGCTGCGAAG CCTTGTCCACCAG
ZZ055-3-F	ATAACAGGTTCAGCAGTCGATGGCC GATGTCGGTGGCG
ZZ055-3-R	TTCACGCCACCGACATCGGCCATCG ACTGCTGAACCTG
ZZ055-4-F	ATAACCGGAATACCTGAAAGAGAA CGCACCGGGCGAAG
ZZ055-4-R	TTCACTTCGCCCGGTGCGTTCTCTTT CAGGTATTCCGG
ZZ072-1-F	ATAACCTCCAAAACAGAGGATACA CTAAATGCAAGAAG
ZZ072-1-R	TTCACTTCTTGCATTTAGTGTATCCTC TGTTTTGGAGG
ZZ072-2-F	ATAACCATCCGGCCTTGGCCAAGTC GGACGCTTCGGCG
ZZ072-2-R	TTCACGCCGAAGCGTCCGACTTGGC CAAGGCCGGATGG
ZZ072-3-F	ATAACCATCTTCCGGAACTCCGGTA ACGCGAACGACGG
ZZ072-3-R	TTCACCGTCGTTCGCGTTACCGGAG TTCCGGAAGATGG
ZZ072-4-F	ATAACTCTATGGATCGAAAGCCTTC CCGCGCCATTCCG
ZZ072-4-R	TTCACGGAATGGCGCGGGAAGGCT TTCGATCCATAGAG
ZZ075-1-F	ATAACACGACGCAAGGGGCGCCCG GAGCGACTTGCATG
ZZ075-1-R	TTCACATGCAAGTCGCTCCGGGCGC CCCTTGCGTCGTG
ZZ075-2-F	ATAACGGCGTTGAGCTTGCGGCCGT TCTCATCGGTCTG
ZZ075-2-R	TTCACAGACCGATGAGAACGGCCG CAAGCTCAACGCCG
ZZ075-3-F	ATAACAACGCCTACGCATGTATCTC GTCGTCAATCAAG

ZZ075-3-R	TTCACTTGATTGACGACGAGATACA TGCGTAGGCGTTG
ZZ075-4-F	ATAACAGTATCGCATCTGGCCGGTC TTCGGGTTCGGCG
ZZ075-4-R	TTCACGCCGAACCCGAAGACCGGC CAGATGCGATACTG
ZZ081-1-F	ATAACGTCTTGTCGCCGCCCGGCTC TGCCGGATCGAAG
ZZ081-1-R	TTCACTTCGATCCGGCAGAGCCGGG CGGCGACAAGACG
ZZ081-2-F	ATAACGGGTCCGTGGCTGATGATCT CGGCAGGAATATG
ZZ081-2-R	TTCACATATTCCTGCCGAGATCATCA GCCACGGACCCG
ZZ081-3-F	ATAACGAAGAGTCTACTGGCCAGAT CGTGGTAAGCCCG
ZZ081-3-R	TTCACGGGCTTACCACGATCTGGCC AGTAGACTCTTCG
ZZ081-4-F	ATAACTTATTGAGGAGCGCACCATG CTGTACATCTGGG
ZZ081-4-R	TTCACCCAGATGTACAGCATGGTGC GCTCCTCAATAAG
ZZ086-1-F	ATAACAACATGGGTCGCAGAGCAG CTTCCGGGTCAACG
ZZ086-1-R	TTCACGTTGACCCGGAAGCTGCTCT GCGACCCATGTTG
ZZ086-2-F	ATAACTGGGGAACCTGGAAGGCGT AACAATCGGGAATG
ZZ086-2-R	TTCACATTCCCGATTGTTACGCCTTC CAGGTTCCCCAG
ZZ086-3-F	ATAACAGGAGAACGTGGAGTTTGCT GACGTATTCCTCG
ZZ086-3-R	TTCACGAGGAATACGTCAGCAAACT CCACGTTCTCCTG
ZZ086-4-F	ATAACTTCAAAAACCGAGGAACCG CCATGAAACTCTAG
ZZ086-4-R	TTCACTAGAGTTTCATGGCGGTTCCT CGGTTTTTGAAG

References

1. Guo, Y.; Chen, P.; Lin, Z.; Wang, T. Characterization of Two Pseudomonas aeruginosa Viruses vB_PaeM_SCUT-S1 and vB_PaeM_SCUT-S2. *Viruses* **2019,** *11*.
2. Lin, Z.; Li, H.; He, L.; Jing, Y.; Pistolozzi, M.; Wang, T.; Ye, Y. Efficient genome editing for Pseudomonas aeruginosa using CRISPR-Cas12a. *Gene* **2021,** *790*, 145693.
3. Klompe, S. E.; Vo, P. L. H.; Halpin-Healy, T. S.; Sternberg, S. H. Transposon-encoded CRISPR-Cas systems direct RNA-guided DNA integration. *Nature* **2019,** *571*, 219-225.
